# Fair Teachers, Fair Police? Assessing the Pathways between Perceptions of Teacher and Police Authority in Childhood and Adolescence

**DOI:** 10.1007/s10964-021-01537-6

**Published:** 2021-11-16

**Authors:** Amy Nivette, Ingrid Obsuth, Denis Ribeaud, Manuel Eisner

**Affiliations:** 1 Department of Sociology, Utrecht University, Utrecht, The Netherlands; 2 Clinical & Health Psychology, University of Edinburgh, Edinburgh, UK; 3 Jacobs Center for Productive Youth Development, University of Zurich, Zurich, Switzerland; 4 Institute of Criminology, University of Cambridge, Cambridge, UK

**Keywords:** Teacher–child relationships, Police legitimacy, Legal socialization, Propensity score matching, Low self-control

## Abstract

Existing research has shown that the quality of the relationship between teacher and child is associated with more positive perceptions of school authorities. There has been relatively less attention to the processes that connect attitudes towards different sources of authority, such as between teachers and police. The current study uses a counterfactual approach to estimate the direct and indirect effects of teacher–child relationships on children’s later perceptions of police legitimacy. Using data from a longitudinal study of youth in Zurich, Switzerland, this study applies non-bipartite propensity score matching to identify matched pairs (*n* = 232 pairs, 55% male) of children with better versus worse relationships with their teacher at age 11 following a teacher change. Matched pairs were then compared on potential mediators (moral norms about deviant behavior and low self-control) at age 13 and perceptions of police legitimacy at age 15. The results demonstrate the importance of the quality of the relationships between students and teachers in shaping young people’s interpersonal characteristics as well as perceptions of the world around them. Namely, if young people feel that they are being treated fairly by their teachers, they are more likely to distinguish behaviors that are right or wrong (moral norms) and control their actions (self-control). Moreover, as a result they are also more likely to perceive authorities such as police as legitimate agents that facilitate societal order.

## Introduction

Early encounters and relationships with school authorities are expected to shape children’s broader understanding of the law and legal authorities (e.g., Mazerolle et al., 2021). Teachers are key actors within this process, as the ways in which teachers treat children and apply rules send signals regarding the trustworthiness and fairness of authorities (e.g., Tyler & Trinkner, 2017). Prior research has shown that the quality of the relationship between teacher and child is associated with more positive perceptions of teacher or school authority (e.g., Caldwell et al., 2021; Gregory & Ripski, 2008; Gouveia-Pereira et al., 2017; Roberson, 2014). However, there has been relatively less attention to the processes that connect attitudes towards different sources of authority, such as between teachers and police (Forrest, 2021). Existing research tends to focus on the correlation between perceptions of nonlegal (parents, teachers) and legal authorities (police, courts), which assumes that youth “generalize” treatment by teachers to police (e.g., Resh, 2018). Building on this research, the current study uses a counterfactual approach to examine to what extent the quality of teacher–child relationships influences children’s later perceptions of police legitimacy. This study also explores whether beliefs about what is right/wrong and the ability to control one’s actions may in part explain this connection between perceptions of teacher and police authority.

### Legal Socialization, Adolescent Development, and the School Context

Legal socialization is defined as the process by which individuals internalize legal norms and develop attitudes towards legal authorities, such as the police (Tapp & Levine, 1977; Trinkner & Reisig, 2021). Indeed, there is growing evidence that this legal socialization process plays a key role in the development of perceptions of police and criminal behavior among adolescents (Cohn et al., 2010; McLean et al., 2018). Legal socialization processes are considered to be age-graded, meaning that individuals encounter different socialization agents and authorities at different stages of the life course (Forrest, 2021). The relative importance of these socialization agents is also expected to change over time. Early in the life course, parents are considered the primary agents of socialization, as they are responsible for providing resources, care, and managing their child’s environment (Grusec, 2011; Sargeant & Bond, 2015; Smetana et al., 2014a; Trinkner et al., 2012). However, by late childhood and early adolescence, children spend more time outside of the home, with the majority of this time spent in school (Eccles & Roeser, 2011).

In the context of development, adolescence is a period of substantial cognitive and social change that enables abstract thinking and critical judgments about the fairness of rules and authorities (Amemiya et al., 2020; Fine et al., 2019). Experiences with school authorities often serve as a child’s first encounter with non-parental rules and punishments (Tyler & Trinkner, 2017). School is also a context in which youth may first experience unfair treatment by institutional authorities (Okonofua et al., 2016). Adolescents are also more open to social influences and sensitive to threats (Granot & Tyler, 2019), meaning that any interactions with school authorities may be particularly pertinent during this developmental period. As such, authorities can have a strong impact on the development and legal socialization and among youth.

Within the school context, teachers may play a role in the legal socialization process in multiple ways. Teachers may increase the likelihood that children adopt and internalize prosocial attitudes and legitimacy beliefs by directly communicating or modeling civic values and norms to their students (Schuitema et al., 2008; Tyler & Trinkner, 2017). Strong bonds and supportive relationships between teachers and children are more likely to lead to the internalization of these values (Maldonado-Carreño & Votruba-Drzal, 2011; Wentzel, 2002). In addition, according to the procedural justice perspective, teachers that enforce rules in a fair, respectful and transparent manner are more likely to be perceived as trustworthy and legitimate (Trinkner & Cohn, 2014). This dynamic can vary between students, as perceptions of teacher authority and fairness differ across children depending on both individual and experiential factors (Murray & Zvoch, 2011; Bayrem Özdemir & Özdemir, 2020; Resh, 2009).

### Direct and Indirect Relations between Perceptions of Teachers and of Police

Questions remain as to how and to what extent these early experiences with nonlegal authorities are associated with attitudes towards legal authorities later in the life course. The majority of research has assessed the direct effects of teacher- or school-related variables on perceptions of police, namely police legitimacy (Ferdik et al., 2014; Fine et al., 2019; Nivette et al., 2021; Trinkner & Cohn, 2014; Wu et al., 2015). These studies tend to assume that experiences with school authorities can be “generalized” to other authorities (Resh & Sabbagh, 2014). This is based on the notion that the quality of interpersonal treatment strengthens group identity and attachment to conventional societal institutions (Tyler & Blader, 2003). Experiences with teachers that are fair and supportive communicate the trustworthiness of authorities associated with these institutions, and evidence suggests that more positive teacher–child relationships and fair treatment are associated with more positive attitudes towards the police and other formal institutions (e.g., Berti et al., 2010; Gouveia-Pereira et al., 2003, 2017; Sanches et al., 2012). Given the correlational nature of these studies, the causal direction of these links or the mechanisms through which they are related are not yet clear.

Available research from education and developmental psychology suggests that teachers may influence outcomes on many dimensions in childhood and adolescent development (McNally & Slutsky, 2018; Sabol & Pianta, 2012). The quality of teacher–child relationships has been associated with a variety of developmental outcomes, including socio-cognitive skills (Eccles & Roeser, 2011), moral beliefs (Nucci, 2001), academic achievement (McCormick & O’Connor, 2015; Wentzel, 2002), and lower aggressive and delinquent behaviors (Obsuth et al., 2017, 2021; O’Connor et al., 2011; Wang et al., 2015). Drawing from this research, this study focuses on two developmental mechanisms that may explain the link between teacher–child relationships and perceptions of police: moral norms about deviant behavior and low self-control. While there are a number of potential mechanisms, moral norms and low self-control are considered to play important roles in shaping how individuals interact with and perceive the police (Nivette et al., 2021; McLean & Wolfe, 2016; Piquero et al., 2004; Reisig et al., 2011). In addition, both mechanisms reflect dispositional characteristics that can be explained using a similar developmental framework. That is, attitudes and behaviors are shaped through individual dispositions and socialization processes characterized by a combination of modeling behaviors, direct discussion, monitoring, and correcting bad behaviors (Grusec, 2011).

#### Teachers and moral norms

Although parents are considered the primary influence on a child’s internalization of moral norms regarding “good” and “bad” behaviors, social domain theory suggests that parents are one of many sources of moral development (Smetana et al., 2014b). Moral norms about deviant behavior are a product of different social interactions with peers, parents, teachers, and other adults (Smetana et al., 2018). Teachers may influence moral norms in the classroom either directly through pedagogical strategies such as mentoring or cooperative learning (Berkowitz, 2011; Buzzelli, 1996; Nucci, 2001), or indirectly though mild discipline coupled with socio-emotional support and appropriate explanation (Grusec et al., 2014). Children must first recognize “virtuous” behavior by teachers and subsequently internalize the underlying values (Willems et al., 2012).

Moral and civic education provides the foundations for children to understand and evaluate the principles of fairness, equality, and voice that ideally underlie legitimate authorities (Tyler & Trinkner, 2017). Internalized moral beliefs about right and wrong contribute to perceptions of moral alignment between individuals and the authorities enforcing the law, considered to be a key component of perceptions of police legitimacy (Jackson, 2018). Related research on political socialization suggests that an open and caring relationship between teachers and children contributes to classroom participation and discussion, and promotes a learning environment conducive to the adoption of civic and moral values (Campbell, 2019; Wanders et al., 2020). More broadly, there is strong evidence that high quality teacher–child relationships are associated with more prosocial and lower aggressive behavior (e.g., Obsuth et al., 2021), which can be related to the evaluation of moral transgressions (Gasser & Keller, 2009; Shavega et al., 2016).

#### Teachers and low self-control

Recent research on police legitimacy has found that attitudes towards the police are associated with individual levels of self-control (e.g., Nivette et al., 2021). Individuals with low self-control tend to seek immediate gratification, engage in risky behaviors, and react in a hostile or defiant manner to authorities and sanctions (Gottfredson & Hirschi, 1990; Piquero et al., 2004; Wolfe, 2011). Studies have shown that individuals with low self-control are more likely to perceive and elicit unfair treatment by authorities (Mastrofski et al., 2002).

Low self-control originates as the result of a combination of biological factors and parental socialization processes, namely effective monitoring and discipline (Botchkovar et al., 2015; Ratchford & Beaver, 2009). There is strong evidence that school authorities also play an active role in socializing youth and teaching self-control (Beaver et al., 2008; Buker, 2011; Gottfredson, 2001; Moon et al., 2014). More positive teacher–child relationships and bonds have likewise been linked to better self-regulation among children in the classroom (Cadima et al., 2016; Rimm-Kaufman et al., 2009). For example, one study examined the relative impact of school compared to family and neighborhood sources of socialization on levels of self-control (Turner et al., 2005). Although parental socialization played a relatively larger role as expected, higher school socialization, measured by the school’s ability to monitor and discipline bad behavior, was associated with higher self-control among adolescents. More broadly, reviews have shown that school and classroom environments characterized by punitiveness, low emotional support, and teacher–child conflict can inhibit the development of executive functioning that regulates impulses, decision-making, and goal-oriented behaviors (Cumming et al., 2020; Vandenbroucke et al., 2018).

Thus, teachers do not only act as models for institutional authority, they may also shape attitudes indirectly by influencing childhood socio-cognitive and moral development. Socio-cognitive dispositions, such as low self-control and internalized moral norms have been found to correlate with later evaluations of police, such as perceptions of procedural justice and legitimacy (Berg et al., 2016; McLean et al., 2018). Early encounters and relationships with nonlegal authorities may therefore relate to later legal attitudes in two ways: first, by providing information about authorities that children can later “generalize” to police, and/or second, by influencing children’s moral and socio-cognitive dispositions, which in turn shape how youth interact with and interpret treatment by police.

#### Issues with causal inference

Importantly, there is a certain developmental order to this process, as children are expected to learn from nonlegal social actors long before their first serious encounter with the police, which typically occurs later in adolescence and adulthood (Fagan & Tyler, 2005). The expectation is therefore that early experiences with nonlegal actors predict attitudes towards the police later in the life course. However, causal evidence of this relationship is limited, as most research relies on cross-sectional surveys or longitudinal research with few time points (see for exceptions, e.g., Trinkner & Cohn, 2014; Mazerolle et al., 2021). Notably, one experimental study provides some causal evidence for the link between experiences with school authority and later perceptions of police legitimacy. In a study conducted in Brisbane, Australia, researchers found that truanting students who participated in a school-based intervention that promoted consensus-based dialog between school, police, parents, and the student were more likely to perceive the police as legitimate compared to those in the control group (Mazerolle et al., 2021). However, the intervention did not have an impact on perceptions of school legitimacy. In addition, it is not clear to what extent changes could be attributed to participation by the police or school authorities within the intervention.

Reliance on cross-sectional and correlational data means that it is not possible to rule out alternative explanations or reverse causation for the association between attitudes towards teachers and police. For example, it is possible that individuals shape the characteristics of their relationships and environments based on their dispositions, known as social selection, or that there is an omitted confounding variable that can account for both perceptions of teachers and police (Jaffree et al., 2012; Nagin & Telep, 2017). One way to approximate causal effects using observational data is to utilize a propensity score matching (PSM) design (Guo & Fraser, 2015).

Propensity score matching techniques can partially account for the limitations of observational data when estimating the causal effects of naturally occurring exogenous “treatment” events (e.g., divorce, school exclusion) on outcomes while controlling for a set of potentially confounding variables (Apel & Sweeten, 2010). In this way, propensity score matching allows researchers to mimic the conditions of randomized controlled trials in order to estimate the treatment effects in a quasi-experimental context (Guo & Fraser, 2015). In essence, it creates equivalent (“balanced”) groups of “treated” vs. “untreated” based on relevant confounding variables, addressing concerns about selection bias.

## The Current Study

The above reviewed literature suggests that teachers can play an important role in the legal socialization process. However, there are two key limitations to existing research. First, existing studies do not adequately account for the social and individual processes that are associated with selection into teacher–child relationships. The current study utilizes non-bipartite propensity score matching to identify matched pairs of children with better versus worse relationships with their teacher at age 11. This matching process can account for factors that determine selection into better or worse relationships and to isolate the impact that teachers have on later attitudes towards the police. Second, little is known about the different ways in which teacher–child relationships are associated with legal attitudes within a developmental context. This study takes an exploratory approach to assessing the direct and indirect relationships between perceptions of teacher and police authority. On the one hand, teacher–child relationships may directly relate to perceptions of police legitimacy, as children interpret experiences with nonlegal authorities as an indication of the trustworthiness of other authorities, including the police. On the other hand, teachers may indirectly play a role in legal socialization through the socialization and internalization of moral norms and self-control. Teacher–child relationships characterized by socio-emotional support and fair treatment are expected to be associated with more prosocial moral norms and higher self-control among adolescents. In turn, prosocial moral norms and self-control are associated with more positive perceptions of police legitimacy.

## Methods

### Participants and Procedure

Data are drawn from 7 waves of the Zurich Project on Social Development from Childhood to Adulthood (z-proso). Z-proso is an ongoing prospective longitudinal cohort study in the City of Zurich that began in 2004 (Eisner et al., 2011). The initial sample included 56 primary schools selected using stratified random sampling, in which disadvantaged school districts were oversampled. The target sample at the initial assessment consisted of 1675 children from these schools (Eisner & Ribeaud, 2005). Active written parental consent was required for the first 6 years of participation in the study, and if the parent did not wish to participate they were asked whether they would allow their child to participate. From wave 5 (age 13), children were legally old enough to give active consent, whereas parents were provided an information letter that allowed them to opt their child out of the study. At ages 13, 15, and 17, participants completed paper and pencil questionnaires in classrooms outside of regular lesson times. At age 20, participants completed surveys on a computer at a university research laboratory. Interviews typically lasted 90 min. Adolescents were compensated for their participation in each wave: ~50 USD at age 15, ~60 USD at age 17, and ~75 USD at age 20. Teachers completed paper and pencil child assessments for each participating child.

In the first three waves (ages ~7, 8, and 9), data were collected from teachers, children, and parents annually. The last data collected from parents occurred when children were aged 11. Data from children were collected biannually from age 11 until age 17 (ages ~11, 13, 15, 17), and again at age 20. Data from teachers continued to be collected annually up until age 13, and then biannually until age 17 (ages ~7, 8, 9, 11, 12, 13, 15, 17). Children typically have the same teacher from grades 1 to 3, and another from grades 4 to 6. After grade 6, children enter a tiered system of secondary schools.

The current study uses data from all three informants at ages 7, 8, and 9 to estimate the propensity score model. The “treatment”, measured by child-reported quality of the teacher–child relationship, was assessed at age 11 and child-reported data collected at age 13 were used to measure the mediating variables moral norms and low self-control. Finally, child-reported perceptions of police legitimacy collected at age 15 was used to measure the outcome. In this way, we are able to establish a plausible temporal order between treatment (X), mediators (M), and outcome (Y), one of the core requirements of causal mediation analysis (Walters & Mandracchia, 2017).

The current analysis only includes cases of students in the present analyses who experienced a teacher change between ages 9 and 10 (1483 students), and for whom data were available related to the child-reported teacher–child relationship. Children who did not experience a teacher change at that age were more likely to have special educational needs and had either completed an extended 2-year first grade or had been retained during the first three years. Of the 1483 children, information about the teacher–child relationship was available from 1067 children. All of them were entered into the matching.

### Measures

#### Police legitimacy

Police legitimacy was first measured at age 15 using three items derived from Sunshine and Tyler (2003) that capture dimensions of police performance, including the quality of treatment and respect (“Police treat people with dignity and respect”), fairness in police decision-making (“Police apply the rules consistently to different people”), and confidence in police effectiveness (“I am confident that the police can do their job well”). Respondents rated their agreement on a four-point Likert scale ranging from “*fully untrue*” to “*fully true*”. Mean scales were calculated (Cronbach’s alpha = 0.81).

#### Moral norms about deviant behavior

The construct of moral norms was used to capture the respondents’ evaluations of rule transgressions and judgments about the wrongfulness of certain deviant behaviors (Wikström and Svensson 2010). The moral norms construct measured at age 13 is an abbreviated scale that refers to five deviant acts: lying to adults, truancy, hitting someone if insulted, stealing something worth less than 5 Swiss Francs (5 USD), and insulting someone out of dislike. These behaviors reflect a range of actions that are considered antisocial across different populations within the context of adolescence (i.e., lying, stealing, aggression) (e.g., Brauer and Tittle 2017; Hirtenlehner and Kunz 2016; Kroneberg et al. 2010). Respondents were asked to record their judgments using a 7-point Likert scale ranging from “*not bad at all*” to “*very bad*”. A mean score was calculated and utilized in the analyses (Cronbach’s alpha = 0.81).

#### Low self-control

Low self-control was also measured at age 13 using 10 items adapted from Grasmick et al. (1993). The abbreviated scale consists of five subdimensions that capture judgment and decision-making processes: impulsivity (e.g., “I often act on the spur of the moment without stopping to think”), self-centeredness (e.g., “If things I do upset people, it’s their problem not mine”), risk-seeking (e.g., “Sometimes I will take a risk just for the fun of it”), preference for physical activities (e.g., “I like to get out and do things more than I like to read or contemplate ideas”), and short temper (e.g., “I lose my temper pretty easily”). Responses are measured on a 4-point Likert scale ranging from “*completely untrue*” to “*completely true*”. A mean score was calculated and utilized in the analyses (Cronbach’s alpha = 0.78).

#### Quality of teacher–child relationship

The “treatment variable” was assessed at age 11 when children reported about their relationship with their teacher by rating the following three statements on a 4-point Likert scale from *completely untrue* to *completely true*: “I get along with my teacher”, “The teacher is fair to me”, and “The teacher supports me”. Cronbach’s alpha was 0.79. A mean score of their responses to these questions was calculated and rounded to an integer yielding a 4-point scale which was utilized as the “treatment” variable in the PSM.

#### Propensity score matching covariates

Covariates in the PSM were 105 variables assessed at ages 7, 8 and 9 that were selected based on their established links to the quality of teacher–child relationships (e.g., Drugli, 2013) and/or because they represented socio-emotional risk factors for a range of negative later outcomes, including low prosociality (e.g., Newton et al., 2014). Specifically, they included five variables per informant (teacher, parent and self) of child behavior and emotions (prosocial behavior, anxiety/depression, attention-deficit hyperactivity disorder, oppositional defiant disorder, and aggressive behavior) measured at ages 7, 8, and 9. Six variables measured child and family characteristics (gender, parents’ migration background, special needs class, parental education, socioeconomic status, and single parent home). Teacher and self-reports of nonaggressive behavior problems and reactive aggression were also included. Four variables assessed parenting based on parent reports and one variable assessed children’s attitude toward homework. Based on teacher-reported data 11 variables assessing school-related constructs were also included, namely school achievement, school cohesion, social role at school, and the parents’ involvement in their child’s school. In addition, child-reported data related to their experiences of bullying victimization, bullying perpetration and observation of bullying at school were also included, as well as the child’s approach to school, whether they liked school and whether they got along with their peers at school. Importantly, one variable per informant assessed the quality of the teacher–child relationship at age 9, that is prior to “treatment”. Finally, two covariates indicated whether the child was the recipient of PATHS and/or Triple P to control for the possible effects of the intervention that was implemented as part of the z-proso project. For further details related to the specific measures, please see Obsuth et al. (2017).

### Analytic procedure

In Zurich, children typically have the same teacher from grades 1 to 3 and another from grades 4 to 6. This study relied on the teacher change, namely the allocation of all children to a new teacher in grade 4 of primary school (at age 10) as a naturally occurring quasi-random assignment to “treatment” in the form of “a teacher with whom a child develops a more positive relationship” (the “treated”) versus “a teacher with whom a child develops a less positive relationship” (the “untreated”).

Optimal non-bipartite matching technique (Lu et al., 2011) was used to find matched pairs of children. Compared to the more commonly utilized bipartite propensity score matching approach that utilizes a dichotomous or dichotomized treatment variable to distinguish between a “treated” and “un-treated” (control) group, the non-bipartite approach takes into account the ordinal scale of the treatment, i.e., the relationship with the teacher in (more than two) different possible “doses”. This allows the consideration of broader variability in the teacher–child relationship. Accordingly, in this study the matched pairs that were identified were as different as possible in their dose of “treatment” (less vs. more positive self-reported teacher–child relationship) yet as similar as possible (not-significantly statistically different) on the covariates, that is on the 105 potentially confounding variables. An ordinal logit model was estimated with the teacher–child relationship as the outcome predicted by the set of 105 covariates to derive a “propensity score” which was utilized to match children (Lu et al., 2011). Matched pairs were required to be within 0.15 standard deviations of the propensity score by applying a caliper (Snodgrasse et al., 2011; for more information on the matching see previous study Obsuth et al., 2017). The matching algorithm generated 284 matched pairs; that is pairs of children who were similar (balanced) on 105 covariates but reported to have a more positive vs. less positive teacher–child relationship. The pairs were checked to ensure that they were balanced on the covariates by carrying out matched samples *t*-test and standardized mean difference statistics (|SMD| < 0.20; Rosenbaum & Rubin, 1985); both of which revealed no significant differences and thus showed that balancing was achieved successfully.

Next, a set of *X*^2^ and *t*-tests were carried out to examine whether the young people who were matched (*n* = 568) based on the teacher–child relationship were different from the rest of the sample with available data (*n* = 499) on a set of demographic variables. The analyses revealed no significant differences between the two groups based on demographic variables, however those that were matched were significantly more likely to have higher teacher-reported prosociality and lower aggression, and were less oppositional at age 7 compared to those who did not enter the matching (see Obsuth et al., 2017: 1669–1670).

Following matching, paired-sample *t*-tests were used as a first step to assess mean differences between the matched pairs of “treated” (more positive teacher–child relationship) versus “untreated” (less positive teacher–child relationship) respondents in police legitimacy at age 15 as well as the mediators at age 13.

However, paired sample *t*-tests do not allow for the assessment of mediation pathways, and so an alternative analytical approach is necessary. In order to estimate the full theoretical model, and calculate direct and indirect effects of teacher–child relationships on police legitimacy, this study used a linear mixed effects approach within a generalized structural equation model [GSEM] framework with the *gsem* command in Stata 16 (StataCorp, 2021). The linear mixed effects model with random intercepts for matched pairs can be considered equivalent to the paired or dependent sample *t*-test (Maxwell et al., 2018). The estimated coefficient for the treatment variable reflects the average difference in the outcome between those in the control (less positive relationships) versus the treatment (more positive relationships) group. Mixed effects GSEMs allow one to simultaneously estimate the direct and parallel mediation pathways while incorporating random effects (i.e., random pair-level intercepts) to account for dependence within the matched pairs. Given that the mediators and outcome variable are continuous and normally distributed, each equation was specified using a Gaussian distribution with identity link function. In the parallel mediation model the mediators were allowed to covary. As recommended by Shrout and Bolger (2002), direct and indirect effects and standard errors were computed using bootstrapping procedures (*n* = 1000 repetitions).

### Missing Data

Listwise deletion was used to ensure that analyses were performed on matched pairs with information on all variables of interest. An analysis of matched participants that were excluded due to listwise deletion showed no significant differences in gender (*M*_included_ = 55.25% male, *M*_excluded_ = 54.63% male), migration background (M_included_ = 39.39% both parents with migrant background, *M*_excluded_ = 40.86% both parents with migrant background), or socio–economic status (*M*_included_ = 50.06, *M*_excluded_ = 49.71). Participants also did not differ on key variables included in the mediation analyses, such as the quality of teacher–child relationship at age 11 (*M*_included_ = 3.34, *M*_excluded_ = 3.26), moral norms (*M*_included_ = 4.49, *M*_excluded_ = 4.46) and low self-control (*M*_included_ = 2.25, *M*_excluded_ = 2.29) at age 13, or police legitimacy at age 15 (*M*_included_ = 2.71, *M*_excluded_ = 2.78). For more general information about attrition between wave 1 (age 7) and wave 7 (age 17) within the study, see Eisner et al., 2019.

## Results

Table 1 presents the overall means and standard deviations for all variables included in the analyses. Table 2 presents the pairwise correlations between all variables used in the analyses.

**Table 1:** Means and standard deviations for the variables included in the analyses

Variable	*N*	*M* (*SD*)	Min	Max
Police legitimacy (age 15)	483	2.721 (0.712)	1	4
Moral norms (age 13)	461	4.490 (1.235)	1	7
Low self-control (age 13)	458	2.256 (0.474)	1	4

Notes. *M* = mean; *SD* = standard deviation

**Table 2:** Pairwise correlations between all variables included in the analyses

	Variable	1	2	3	4
1	Better relationship (age 11) (Ref: worse relationship)	1			
2	Moral norms (age 13)	0.105*	1		
3	Low self-control (age 13)	−0.169***	−0.408***	1	
4	Police legitimacy (age 15)	0.128*	0.218***	−0.249***	1

Notes. *p < 0.05; ***p < 0.001

### Direct Effects

The effect of teacher–child relationship at age 11 on perceptions of police legitimacy at age 15 is reported in Table 3. The results show that young people who reported a better relationship with their teacher at age 11 were significantly more likely to perceive the police as legitimate at age 15 (*t* = −2.897, *p* = 0.004). The size of the effect can be considered ‘small’ (Cohen’s *d* = −0.189, 95%CI = −0.371, −0.007). Table 3 also shows that a better relationship with one’s teacher was associated with significantly higher adherence to moral norms (*t* = −2.188, *p* = 0.030) and higher self-control (*t* = 3.636, *p* < 0.001) at age 13. The effect sizes for moral norms was small (Cohen’s *d* = 0.097, 95%CI = −0.342, 0.040), whereas the effect size for low self-control was relatively larger (Cohen’s *d* = 0.252, 95%CI = 0.059, 0.445).

**Table 3:** Mean differences between matched pairs based on the quality of teacher–child relationship at age 11 for police legitimacy at age 15 and mediator variables at age 13

Age	Outcome	*N* pairs	Better rel. *M*(*SD*)	Worse rel. *M*(*SD*)	Paired sample *t*-test	*p*-value
15	Police legitimacy	232	2.812 (0.668)	2.624 (0.748)	−2.879	0.004
	*Mediating variables*					
13	Moral norms	210	4.617 (1.301)	4.358 (1.162)	−2.188	0.030
	Low self-control	208	2.166 (0.471)	2.330 (0.471)	3.636	<0.001

### Mediation Pathways

Table 4 displays the results for the GSEMs estimating the parallel mediation pathways connecting teacher–child relationship (X), moral norms (M1), low self-control (M2), and police legitimacy (Y). In line with the paired sample *t*-tests, children who reported better relationships with their teachers reported more prosocial moral norms (*b* = 0.261, *SE* = 0.123, *p* = 0.034) and higher self-control (*b* = −0.160, *SE* = −0.047, *p* = 0.001) at age 13. In turn, youth who scored higher on the low self-control scale had more negative perceptions of police legitimacy at age 15 (*b* = −0.276, *SE* = 0.081, *p* = 0.001). Youth with higher adherence to moral norms reported higher levels of police legitimacy at age 15 (*b* = 0.078, *SE* = 0.031, *p* = 0.011). The direct pathway from teacher–child relationships at age 11 to police legitimacy at age 15 was no longer significant (*b* = 0.122, *SE* = 0.070, *p* = 0.081).

**Table 4:** Generalized structural equation models estimating the effects of the quality of teacher–child relationship at age 11 on mediating variables at age 13 and police legitimacy at age 15 (*n* = 200 pairs)

	*b/SE*	95% LCI	95% UCI	*p*-value
		DV: low self-control (age 13)		
Better relationship (age 11)	−0.160	−0.251	−0.069	0.001
(Ref: worse relationship)	0.047			
Constant	2.331	2.267	2.396	<0.001
	0.033			
Variance (pair level)	0.001			
	0.013			
Variance (individual level)	0.217			
	0.020			
		DV: moral norms (age 13)		
Better relationship (age 11)	0.261	0.020	0.502	0.034
(Ref: worse relationship)	0.123			
Constant	4.363	4.191	4.535	<0.001
	0.088			
Variance (pair level)	0.029			
	0.093			
Variance (individual level)	1.514			
	0.141			
		DV: police legitimacy (age 15)		
Better relationship (age 11)	0.122	−0.015	0.259	0.081
(Ref: worse relationship)	0.070			
Low self-control (age 13)	−0.276	−0.435	−0.116	0.001
	0.081			
Moral norms (age 13)	0.078	0.018	0.139	0.011
	0.031			
Constant	2.917	2.374	3.459	<0.001
	0.177			
Variance (pair level)	0.008			
	0.035			
Variance (individual level)	0.476			
	0.048			
Covariance (moral norms*low self-control)	−0.231	−0.292	−0.169	<0.001
	0.031			

Notes. Unstandardized coefficients are reported. DV = dependent variable; *SE* = standard error; LCI = lower 95% confidence interval; UCI = upper 95% confidence interval. *N* observations = 400, *N* pairs = 200

The indirect effects for each pathway and total effects with bootstrapped standard errors are presented in Table 5. The results suggest that while individual pathways connecting the quality of teacher–child relationships and moral norms, and moral norms and police legitimacy were significant in the GSEM model (Table 4), the indirect effect via moral norms was not significantly different from zero by conventional thresholds (*b* = 0.020, *SE* = 0.012, *p* = 0.080). The indirect pathway connecting teacher–child relationships to police legitimacy via low self-control is positive and significant (*b* = 0.044, *SE* = 0.020, *p* = 0.030). Taken together, the total indirect effect was also positive and significant (*b* = 0.065, *SE* = 0.022, *p* = 0.003).

**Table 5:** Indirect and total effects of the quality of teacher–child relationship on mediators at age 13 and police legitimacy at age 15

Pathway	*b/SE*	95% LCI	95% UCI	*p*-value
		*Indirect effects*		
Better relationship → moral norms	0.020	−0.002	0.043	0.080
	0.012			
Better relationship → low self-control	0.044	0.004	0.084	0.030
	0.020			
Total indirect effect	0.065	0.022	0.108	0.003
	0.022			
		*Total effects*		
Better relationship → police legitimacy	0.187	0.049	0.324	0.008
	0.070			

Notes. SE = standard error; LCI = lower confidence interval; UCI = upper confidence interval. Standard errors are calculated using bootstrapping techniques with 1000 repetitions

An additional set of sensitivity analyses were conducted including controls for prior low self-control (age 11) and teacher–child relationships (age 13). The results are generally robust to the addition of these control variables (more information and full results of the sensitivity analyses are presented in the Appendix).

## Discussion

Early relationships with school authorities can shape later attitudes towards the law and the police, however less is known about the developmental pathways that connect attitudes towards teachers and police. Using propensity score matching in a quasi-experimental context, this study examined the direct and indirect pathways connecting teacher–child relationships at age 11 and attitudes towards the police at age 15. Overall, the findings suggest that better and more fair relationships with teachers in late childhood are associated with more positive attitudes towards the police during adolescence. This lends support to the notion that relationships and encounters with nonlegal authorities early in the life course form the basis for how youth later interact with and perceive legal authorities (e.g., Cardwell et al., 2021).

Furthermore, this research expands on and integrates legal socialization and developmental frameworks to assess how socialization agents might play a role in socio-cognitive development, and in turn perceptions of police legitimacy. While previous research has highlighted the importance of individual dispositions and socio-cognitive characteristics in shaping legal attitudes (Ameri et al., 2019; Jackson et al., 2020), few have specified and tested these integrated theoretical pathways (e.g., Augustyn & Ray, 2016). The results suggest that early relationships with nonlegal socialization agents also play a role in the development of moral norms and self-control, which are in turn jointly associated with more positive perceptions of police legitimacy. This implies that the processes contributing to legal socialization operate more broadly during child development, as the quality of relationships with teachers also contribute to individual values and dispositions that determine how adolescents perceive and interact with rules and authorities. Given that moral norms and low self-control are also strong predictors of delinquent behavior (Herman & Pogarsky, 2020; Kronenberg et al., 2010; Vazsonyi et al., 2017), early encounters and relationships with teachers that act in a fair, open, and respectful manner may also have long-term preventive effects on both criminal and legal attitudinal outcomes.

It is important to note that with the addition of mediators, the direct effect of relationships on police legitimacy was no longer significant. This seems to suggest that attitudes towards teacher authority may not directly generalize to police authorities. However, it is still possible that youth also generalize experiences across authorities, since police legitimacy was only first measured at age 15. Ideally, perceptions of police legitimacy should also be measured at age 13 in order to estimate the strength of the direct “generalized” pathway against the indirect pathways. In addition, the current study only focused on the connection between teacher and police authorities, whereas previous research has shown that relationships with parents, peers, and other authorities during adolescence may be associated with attitudes towards the police (e.g., Cavanaugh & Cauffman, 2015; Granot et al., 2021; Kaiser & Reisig, 2017). Future research should examine whether and to what extent these experiences generalize to one another, and how do these effects play out over the life course.

In addition, due in part to the small sample size, this study explored only two potential dispositional characteristics that theoretically link teacher–child relationships and perceptions of police legitimacy. More research is needed to examine other attitudinal and behavioral mediating mechanisms that explain how early relationships with nonlegal authorities are associated with perceptions of police legitimacy among youth. For example, within and outside the school context, adolescents are also subject to peer influences that can shape their moral norms and motivations (Malti & Buchmann 2010), sensitivity to (in)justice (Jones & Skarlicki, 2005), and self-control (Meldrum & Hay, 2012). The peer context in which adolescents are embedded in- and outside the classroom may therefore determine how youth develop relationships with teachers and react to their authority (Fine et al., 2016; Young & Rees, 2013). In addition, less positive teacher–child relationships have been shown to predict later delinquent and aggressive behaviors (see e.g., Obsuth et al., 2021), which may increase the likelihood that youth come in contact with the police. If the quality of the treatment during these contacts is perceived to be poor, this can result in more negative perceptions of police legitimacy (Augustyn, 2016; Maguire et al., 2017; Walters & Bolger, 2018). Delinquent behavior may also be directly related to perceptions of police, as youth develop negative attitudes to justify rule-breaking and wrongdoing (Nivette et al., 2021). In childhood and early adolescence, the role of parents is also important to consider in conjunction with teachers in the socialization of legal attitudes (Sindall et al., 2017; Trinkner et al., 2012; Wolfe et al., 2017). Research on political socialization shows that civic education in schools can “compensate” for differences in parental socialization (Neundorf et al., 2016). Additional research is necessary to determine how complementing and competing informal and formal socialization agents interact to shape legal attitudes over the life course.

There are several limitations to the current study. First, while the study is strengthened by the use of prospective longitudinal data spanning 13 years, the reliance on existing data meant that some measures of concepts were less than optimal. The measure of teacher–child relationship consisted of three items, capturing elements of fairness, support, and attachment. Similarly, the measure of police legitimacy consists of three items including fair treatment and trustworthiness. Ideally, measures of authority should include processed-based judgments regarding treatment by teachers and police, as well as related concepts of distributive justice, moral alignment, and legality (Jackson & Gau, 2016; Tankebe, 2013). In order to more precisely assess socialization processes, researchers should include different mechanisms of socialization such as socio-emotional bonds, monitoring, and discipline (Grusec, 2011). Additionally, there is some debate over the construct validity of the Grasmick et al., (1993) low self-control scale and whether self-report measures are adequate for capturing self-control (Walters, 2016). Future studies should assess the robustness of the results using different behavioral, self- and informant-reported operationalizations of self-control (Duckworth & Kern, 2011).

Second, although the current study included a large number of covariates (105) in the matching process, it is possible that there are unmeasured confounds that can impact the results. In particular, while factors were included that can account for general prosociality, attitudes specific to police legitimacy prior to age 11 were not included. More generally, there is little understanding of the emergence, continuity, and change in legal attitudes among young children. Legal socialization research would greatly benefit from studies utilizing “child-friendly” questionnaires or alternative participatory techniques (see e.g., Fargas-Malet et al., 2010) to measure and assess attitudes towards the police starting in early childhood.

## Conclusion

The current study demonstrates the importance of early relationships with teachers in shaping perceptions of police legitimacy, and the interconnection between perceptions of nonlegal and legal authorities within the legal socialization process. In particular, propensity score matching techniques were utilized to overcome previous methodological limitations to estimate the long-term relationship between teacher authority and perceptions of the police. Taken together, the findings reinforce the importance of legal socialization, specifically the role of the quality of teacher–child relationships in fostering positive developmental outcomes and legal orientations. Controlling for a large number of other factors, such as parenting, problem behaviors, and mental health problems that may potentially influence young people’s development, our findings highlight the importance of the quality of the relationships between students and teachers in shaping young people’s interpersonal characters as well as perceptions of the world around them. Namely, if young people feel that they are being treated fairly by their teachers, they are more likely to distinguish behaviors that are right or wrong (moral norms) and control their actions (self-control). Moreover, as a result they are also more likely to perceive authorities, such as police, as legitimate agents that facilitate societal order. These links are particularly relevant in adolescence given that this is the developmental period during which young people become more open and susceptible to broader societal influences and threats. It is important that teacher training programs incorporate instruction on teachers’ understanding of the impact they have on young people’s perceptions of authorities at school and beyond, as well as foster teachers’ abilities to develop high quality relationships with students that would enhance their credibility as authorities with their best interest in mind.

## Appendix

**A. Additional Analyses**

The following analyses include additional controls for prior low self-control (age 11) and teacher–child relationships (age 13). Unfortunately, the construct of moral norms about deviant behavior was only first measured at age 13, and so we were not able to include this in the additional analyses. However, when the additional mediation and control variables were added into the structural equation model, the model would not converge. This is likely due to the relatively low sample size of matched pairs (*n* = 200 matched pairs). Nevertheless, we can assess the basic relationships using multilevel mixed models for available measures. Although multilevel mixed models cannot estimate full mediation pathways, it can give a sense of the associations when controlling for key variables.

The results presented below in Tables 6 and 7 suggest that the relationship between teacher–student relationships and low self-control is slightly reduced in size but remains significant when a prior measure of low self-control (age 11) is included in the model. Similarly, Table 7 shows that the size of the associations between moral norms (age 13), low self-control (age 13) and police legitimacy (age 15) are somewhat reduced, but remain significant at the 0.05 level.

**Table 6:** Mixed effects model estimating effects of teacher–child relationship at age 11 on low self-control at age 13 controlling for low self-control at age 11 (*n* = 199 pairs)

Variables	*b*/SE	95% LCI	95% UCI	*p*-value
Better relationship (age 11)	−0.104	−0.19	−0.018	0.017
(Ref: worse relationship)	0.043			
Low self-control (age 11)	0.373	0.281	0.464	<0.001
	0.467			
Constant	1.563	1.365	1.761	<0.001
	0.101			
Variance (pair level)	0.002			
	0.013			
Variance (individual level)	0.185			
	0.019			

Notes. Unstandardized coefficients are reported. SE = standard error; LCI = lower 95% confidence interval; UCI = upper 95% confidence interval. N observations = 398, N pairs = 199

**Table 7:** Mixed effects model estimating effects of teacher–child relationship at age 11 on police legitimacy at age 15 controlling for teacher–child relationship at age 13 (*n* = 200 pairs)

Variables	*b*/SE	95% LCI	95% UCI	*p*-value
Better relationship (age 11)	0.010	−0.037	0.236	0.151
(Ref: worse relationship)	0.070			
Low self-control (age 13)	−0.215	−0.38	−0.051	0.010
	0.084			
Moral norms (age 13)	0.061	0.001	0.122	0.047
	0.031			
Teacher–child relationship (age 13)	0.155	0.036	0.274	0.010
	0.06			
Constant	2.381	1.704	3.058	<0.001
	0.345			
Variance (pair level)	0.015			
	0.034			
Variance (individual level)	0.462			
	0.047			

Notes. Unstandardized coefficients are reported. SE = standard error; LCI = lower 95% confidence interval; UCI = upper 95% confidence interval. N observations = 400, N pairs = 200

## References

[CR1] Amemiya J, Fine A, Wang M-T (2020). Trust and discipline: Adolescents’ institutional and teacher trust predict classroom behavioral engagement following teacher discipline. Child Development.

[CR2] Ameri T, Burgason KA, DeLisi M, Heirigs MH, Hochstetler A, Vaughn MG (2019). Legal cynicism: Independent construct or downstream manifestation of antisocial constructs? New evidence. International Journal of Law and Psychiatry.

[CR3] Apel RJ, Sweeten G, Piquero AR, Weisburd D (2010). Propensity score matching in criminology and criminal justice. Handbook of Quantitative Criminology.

[CR4] Augustyn MB (2016). Updating perceptions of (in)justice. Journal of Research in Crime and Delinquency.

[CR5] Augustyn MB, Ray JV (2016). Psychopathy and perceptions of procedural justice. Journal of Criminal Justice.

[CR6] Beaver KM, Wright JP, Maume MO (2008). The effect of school classroom characteristics on low self-control: A multilevel analysis. Journal of Criminal Justice.

[CR7] Bayrem Özdemir S, Özdemir M (2020). How do adolescents’ perceptions of relationships with teachers change during upper-secondary school years?. Journal of Youth and Adolescence.

[CR8] Berg MT, Stewart EA, Intravia J, Warren P, Simons RL (2016). Cynical streets: Neighborhood social pro- cesses and perceptions of criminal injustice. Criminology.

[CR9] Berkowitz MW (2011). What works in values education. International Journal of Educational Research.

[CR10] Berti C, Molinari L, Speltini G (2010). Classroom justice and psychological engagement: Students’ and teachers’ representations. Social Psychology of Education.

[CR11] Botchkovar E, Marshall IH, Rocque M, Posick C (2015). The importance of parenting in the development of self-control in boys and girls: Results from a multinational study of youth. Journal of Criminal Justice.

[CR12] Brauer JR, Tittle CR (2017). When crime is not an option: Inspecting the moral filtering of criminal action alternatives. Justice Quarterly.

[CR13] Buker H (2011). Formation of self-control: Gottfredson and Hirschi’s general theory of crime and beyond. Aggression and Violent Behavior.

[CR14] Buzzelli CA (1996). The moral implications of teacher-child discource in early childhood classrooms. Early Childhood Research Quarterly.

[CR15] Cadima J, Verschueren K, Leal T, Guedes C (2016). Classroom interactions, dyadic teacher-child relationships, and self-regulation in socially disadvantaged young children. Journal of Abnormal Child Psychology.

[CR16] Campbell DE (2019). What social scientists have learned about civic education: A review of the literature. Peabody Journal of Education.

[CR17] Cardwell SM, Mazerolle L, Piquero AR, Luengen K (2021). Perceiving teachers as procedurally just limits the negative effects of delinquent peer associations: An analysis of Australian adolescent boys’ and girls’ perceptions of school authority. Journal of Social Issues.

[CR18] Cavanaugh C, Cauffman E (2015). Viewing law and order: Mothers’ and sons’ justice system legitimacy attitudes and juvenile recidivism. Psychology, Public Policy, and Law.

[CR19] Cohn ES, Bucolo D, Rebellon CJ, van Gundy K (2010). An integrated model of legal and moral reasoning and rule-violating behavior: The role of legal attitudes. Law & Human Behavior.

[CR20] Cumming MM, Bettini E, Pham AV, Park J (2020). School-, classroom-, and dyadic-level experiences: A literature review of their relationship with students’ executive functioning development. Review of Educational Research.

[CR21] Drugli MB (2013). How are closeness and conflict in student–teacher relationships associated with demographic factors, school functioning and mental health in Norwegian schoolchildren aged 6–13?. Scandinavian Journal of Educational Research.

[CR22] Duckworth AL, Kern ML (2011). A meta-analysis of the convergent validity of self-control measures. Journal of Research in Personality.

[CR23] Eccles JS, Roeser RW (2011). Schools as developmental contexts during adolescence. Journal of Research on Adolescence.

[CR25] Eisner M, Malti T, Ribeaud D, Gadd D, Karstedt S, Messner S (2011). Large-scale criminological field experiments: The Zurich project on the social development of children. The SAGE handbook of criminological research methods.

[CR26] Eisner M, Ribeaud D (2005). A randomised field experiment to prevent violence: The Zurich intervention and prevention project at schools, ZIPPS. European Journal of Crime, Criminal Law and Criminal Justice.

[CR125] Eisner N, Murray AL, Eisner, M, Ribeaud D (2019). A practical guide to the analysis of non-response and attrition in longitudinal research using a real data sample. International Journal of Behavioral Development.

[CR27] Fagan J, Tyler TR (2005). Legal socialization of children and adolescents. Social Justice Research.

[CR28] Fargas-Malet M, McSherry D, Larkin E, Robinson C (2010). Research with children: Methodological issues and innovative techniques. Journal of Early Childhood Research.

[CR29] Ferdik FV, Wolfe SE, Blasco N (2014). Informal social controls, procedural justice and perceived police legitimacy: Do social bonds influence evaluations of police legitimacy?. American Journal of Criminal Justice.

[CR30] Fine A, Cavanagh C, Donley S, Steinberg L, Frick PJ, Cauffman E (2016). The role of peer arrests on the development of youths’ attitudes towards the justice system. Law and Human Behavior.

[CR31] Fine AD, Kan E, Cauffman E (2019). Adolescents’ confidence in institutions: Do America’s youth differentiate between legal and social institutions?. Developmental Psychology.

[CR33] Forrest, W. (2021). The contribution of intimate relationships to legal socialization: Legitimacy, legal cynicism, and relationship characteristics. *Journal of Social Issues*, 10.1111/josi.12438.

[CR34] Gasser L, Keller M (2009). Are the competent the morally good? Perspective taking and moral motivation of children involved in bullying. Social Development.

[CR35] Gottfredson DC (2001). Schools and delinquency.

[CR36] Gottfredson MR, Hirschi T (1990). A general theory of crime.

[CR37] Gouveia-Pereira M, Vala J, Correia I (2017). Teachers’ legitimacy: Effects of justice perception and social comparison processes. British Journal of Educational Psychology.

[CR38] Gouveia-Pereira M, Vala J, Palmonari A, Rubini M (2003). School experience, relational justice and legitimation of institutional. European Journal of Psychology of Education.

[CR39] Granot Y, Tyler TR (2019). Adolescent cognition and procedural justice: Broadening the impact of research findings on policy and practice. Social and Personality Psychology Compass.

[CR40] Granot, Y., Tyler, T.R., & Durkin, A. (2021). Legal socialization during adolescence: The emerging role of school resource officers. *Journal of Social Issues*, 10.1111/josi.12446.

[CR41] Grasmick HG, Tittle CR, Bursik RJ, Arneklev BJ (1993). Testing the core empirical implications of Gottfredson and Hirschi’s general theory of crime. Journal of Research in Crime and Delinquency.

[CR42] Gregory A, Ripski M (2008). Adolescent trust in teachers: Implications for behavior in the high school classroom. School Psychology Review.

[CR43] Grusec JE (2011). Socialization processes in the family: Social and emotional development. Annual Review of Psychology.

[CR44] Grusec J, Chaparro MP, Johnston M, Sherman A, Killen M, Smetana JG (2014). The development of moral behavior from a socialization perspective. Handbook of moral development.

[CR45] Guo S, Fraser MW (2015). Propensity score analysis: Statistical methods and applications.

[CR46] Herman, S. & Pogarsky, G. (2020). Morality, deterrability, and offender decision making. *Justice Quarterly*, 10.1080/07418825.2019.1709884.

[CR47] Hirtenlehner H, Kunz F (2016). The interaction between self-control and morality in crime causation among older adults. European Journal of Criminology.

[CR48] Jackson DB, Testa A, Vaughn MG (2020). Low self-control and legal cynicism among at-risk youth: An investigation into direct and vicarious police contact. Journal of Research in Crime and Delinquency.

[CR49] Jackson J (2018). Norms, Normativity, and the Legitimacy of Justice Institutions: International Perspectives. Annual Review of Law and Social Science.

[CR50] Jackson J, Gau J, Shockley E, Neal TMS, PytlikZillig LM, Bornstein BH (2016). Carving up concepts? Differentiating between trust and legitimacy in public attitudes towards legal authority. Interdisciplinary perspectives on trust: Towards theoretical and methodological integration.

[CR51] Jaffee SR, Strait LB, Odgers CL (2012). From correlates to causes: can quasi-experimental studies and statistical innovations bring us closer to identifying the causes of antisocial behavior?. Psychological bulletin.

[CR52] Jones DA, Skarlicki DP (2005). The effects of overhearing peers discuss an authority’s fairness reputation on reactions to subsequent treatment. Journal of Applied Psychology.

[CR53] Kaiser, K., & Reisig, M. D. (2017). Legal socialization and self-reported criminal offending: The role of procedural justice and legal orientations. *Journal of Quantitative Criminology*, 10.1007/s10940-017-9375-4

[CR54] Kronenberg C, Heintze I, Mehlkop G (2010). The interplay of moral norms and instrumental incentives in crime causation. Criminology.

[CR56] Lu B, Greevy R, Xu X, Beck C (2011). Optimal nonbipartite matching and its statistical applications. The American Statistician.

[CR57] Maguire ER, Lowrey BV, Johnson D (2017). Evaluating the relative impact of positive and negative encounters with the police: A randomized experiment. Journal of Experimental Criminology.

[CR58] Maldonado-Carreño C, Votruba-Drzal E (2011). Teacher-child relationships and the development of academic and behavioral skills during elementary school: A within- and between-child analysis. Child Development.

[CR59] Malti T, Buchmann M (2010). Socialization and individual antecedents of adolescents’ and young adults’ moral motivation. Journal of Youth and Adolescence.

[CR60] Mastrofski SD, Reisig MD, McCluskey JD (2002). Police disrespect toward the public: An encounter-based analysis. Criminology.

[CR61] Maxwell SE, Delaney HD, Kelley K (2018). Designing experiments and analyzing data: A model comparison perspective.

[CR62] Mazerolle L, Antrobus E, Cardwell SM, Piquero AR, Bennett S (2021). Harmonizing legal socialization to reduce antisocial behavior: Results from a randomized field trial of truanting young people. Justice Quarterly.

[CR63] McCormick MP, O’Connor EE (2015). Teacher-child relationship quality and academic achievement in elementary school: Does gender matter?. Journal of Educational Psychology.

[CR64] McLean K, Wolfe SE (2016). A sense of injustice loosens the moral bind of the law: Specifying the links between procedural injustice, neutralizations, and offending. Criminal Justice and Behavior.

[CR65] McLean, K., Wolfe, S. E., & Pratt, T. C. (2018). Legitimacy and the life course: An age-graded examination of changes in legitimacy attitudes over time. *Journal of Research in Crime and Delinquency*, 10.1177/0022427818793934

[CR66] McNally S, Slutsky R (2018). Teacher-child relationships make all the difference: constructing quality interactions in early childhood settings. Early Childhood Development and Care.

[CR67] Meldrum RC, Hay C (2012). Do peers matter in the development of self-control? Evidence from a longitudinal study. Journal of Youth and Adolescence.

[CR68] Moon B, McClusky JD, Blurton D, Hwang HW (2014). Parent and teacher practices as sources of low self-control: Evidence from Korea. Youth Violence and Juvenile Justice.

[CR69] Murray C, Zvoch K (2011). Teacher-student relationships among behaviorally at-risk African American youth from low-income backgrounds: Student perceptions, teacher perceptions, and socioemotional adjustment correlates. Journal of Emotional and Behavioral Disorders.

[CR70] Nagin DS, Telep CW (2017). Procedural justice and legal compliance. Annual Review of Law and Social Science.

[CR71] Neundorf A, Niemi RG, Smets K (2016). The compensation effect of civic education on political engagement: How civics classes make up for missing parental socialization. Political Behavior.

[CR72] Newton EK, Laible D, Carlo G, Steele JS, McGinley M (2014). Do sensitive parents foster kind children, or vice versa? Bidirectional influences between children’s prosocial behavior and parental sensitivity. Developmental Psychology.

[CR73] Nivette A, Eisner M, Ribeaud D (2021). Evaluating the shared and unique predictors of legal cynicism and police legitimacy from adolescence into early adulthood. Criminology.

[CR74] Nucci LP (2001). Education in the Moral Domain.

[CR75] Obsuth, I., Murray, A.L., Knoll, M., Ribeaud, D., & Eisner, M. (2021). Teacher-student relationships in childhood as a protective factor against adolescent delinquency up to age 17: A propensity score matching approach. *Crime & Delinquency*, 10.1177/00111287211014153. 10.1177/00111287211014153 PMC10026349 36960348

[CR76] Obsuth I, Murray AL, Malti T, Sulger P, Ribeaud D, Eisner M (2017). A non-bipartite propensity score analysis of the effects of teacher–student relationships on adolescent problem and prosocial behavior. Journal of Youth and Adolescence.

[CR77] O’Connor E, Dearing E, Collins BA (2011). Teacher-child relationship and behavior problem trajectories in elementary school. American Educational Research Journal.

[CR78] Okonofua JA, Walton GM, Eberhardt JL (2016). A vicious cycle: A social-psychological account of extreme racial disparities in school discipline. Perspectives on Psychological Science.

[CR79] Piquero AR, Gomez-Smith Z, Langton L (2004). Discerning unfairness where others may not: Low self-control and unfair sanction perceptions. Criminology.

[CR80] Ratchford M, Beaver KM (2009). Neuropsychological deficits, low self-control, and delinquent involvement: Toward a biosocial explanation of delinquency. Criminal Justice and Behavior.

[CR81] Reisig MD, Wolfe SE, Holtfreter K (2011). Legal cynicism, legitimacy, and criminal offending: The nonconfounding effect of low self-control. Criminal Justice and Behavior.

[CR82] Resh N (2018). Sense of justice in school and social and institutional trust. Comparative Sociology.

[CR83] Resh N (2009). Justice in grades allocation: teachers’ perspective. Social Psychology of Education.

[CR84] Resh N, Sabbagh C (2014). Justice, belonging and trust among Israeli middle school students. British Educational Research Journal.

[CR86] Rimm-Kaufman SE, Grimm KJ, Curby TW, Nathanson L, Brock LL (2009). The contribution of children’s self-regulation and classroom qualities to children’s adaptive behaviors in the kindergarten classroom. Developmental Psychology.

[CR87] Roberson RR (2014). Understanding the Development of Legitimate Teacher Authority through the Teacher–Student Relationship: A Qualitative Study. Unpublished doctoral diss..

[CR88] Rosenbaum PR, Rubin DB (1985). Constructing a control group using multivariate matched sampling methods that incorporate the propensity score. The American Statistician.

[CR89] Sabol TJ, Pianta RC (2012). Recent trends in research on teacher-child relationships. Attachment & Human Development.

[CR90] Sanches C, Gouveia-Pereira M, Carugati F (2012). Justice judgements, school failure, and adolescent deviant behavior. British Journal of Educational Psychology.

[CR91] Sargeant E, Bond CEW (2015). Keeping it in the family: Parental influences on young people’s attitudes to police. Journal of Sociology.

[CR92] Schuitema J, ten Dam G, Veugelers W (2008). Teaching strategies for moral education: A review. Journal of Curriculum Studies.

[CR93] Shavega TJ, van Tuijl C, Brugman D (2016). Aggressive and prosocial children’s evaluation and justification of transgressions and their relationship to the teacher-child relationship in Tanzania. Early Childhood Research Quarterly.

[CR94] Shrout PE, Bolger N (2002). Mediation in experimental and nonexperimental studies: New procedures and recommendations. Psychological Methods.

[CR95] Sindall K, McCarthy DJ, Brunton-Smith I (2017). Young people and the formation of attitudes towards the police. European Journal of Criminology.

[CR96] Smetana JG, Jambon M, Ball C (2018). Normative changes and individual differences in early moral judgments: a constructivist developmental perspective. Human Development.

[CR97] Smetana JG, Robinson J, Rote WM, Grusec JE, Hastings PD (2014). Socialization in adolescence. Handbook of Socialization.

[CR98] Smetana JG, Jambon M, Ball C, Killen M, Smetana JG (2014). The social domain approach to children’s moral and social judgments. Handbook of moral development.

[CR99] Snodgrasse GM, Blokland AA, Haviland A, Nieuwbeerta P, Nagin DS (2011). Does the time cause the crime? An examination of the relationship between time served and reoffending in the Netherlands. Criminology.

[CR100] StataCorp. (2021). Stata Statistical Software: Release 17.

[CR101] Sunshine J, Tyler TR (2003). The role of procedural justice and legitimacy in shaping public support for policing. Law & Society Review.

[CR102] Tankebe J (2013). Viewing things differently: The dimensions of public perceptions of police legitimacy. Criminology.

[CR103] Tapp JL, Levine FJ (1977). Law, justice, and the individual in society: Psychological and legal issues.

[CR104] Trinkner R, Cohn ES (2014). Putting the “social” back in legal socialization: Procedural justice, legitimacy, and cynicism in legal and nonlegal authorities. Law and Human Behavior.

[CR105] Trinkner R, Cohn ES, Rebellon CJ, Van Gundy K (2012). Don’t trust anyone over 30: Parental legitimacy as a mediator between parenting style and changes in delinquent behavior over time. Journal of Adolescence.

[CR106] Trinkner, R. & Reisig, M.D. (2021). Celebrating 50 years of legal socialization. *Journal of Social Issues*, 10.1111/josi.12458.

[CR107] Turner MG, Piquero AR, Pratt TC (2005). The school context as a source of self-control. Journal of Criminal Justice.

[CR108] Tyler TR, Blader SL (2003). The group engagement model: Procedural justice, social identity, and cooperative behavior. Personality and Social Psychology Review.

[CR109] Tyler TR, Trinkner R (2017). Why children follow rules: Legal socialization and the development of legitimacy.

[CR110] Vandenbroucke L, Split J, Verschueren K, Piccinin C, Baeyens D (2018). The classroom as a developmental context for cognitive development: A meta-analysis on the importance of teacher-student interactions for children’s executive functions. Review of Educational Research.

[CR111] Vazsonyi AT, Mikuška J, Kelley EL (2017). It’s time: A meta-analysis on the self-control-deviance link. Journal of Criminal Justice.

[CR112] Walters GD (2016). Are behavioral measures of self-control and the Grasmick self-control scale measuring the same construct? A meta-analysis. American Journal of Criminal Justice.

[CR113] Walters, G. D., & Bolger, P. C. (2018). Procedural justice perceptions, legitimacy beliefs, and compliance with the law: A meta-analysis. *Journal of Experimental Criminology*, Epub ahead of print. 10.1007/s11292-018-9338-2

[CR114] Walters GD, Mandracchia JT (2017). Testing criminological theory through causal mediation analysis: Current status and future directions. Journal of Criminal Justice.

[CR115] Wanders FHK, Dijkstra AB, Maslowski R, van der Veen I (2020). The effect of teacher-student and student-student relationships on the societal involvement of students. Research Papers in Education.

[CR116] Wang C, Swearer SM, Lembeck P, Collins A, Berry B (2015). Teachers matter: An examination of student-teacher relationships, attitudes towards bullying, and bullying behavior. Journal of Applied School Psychology.

[CR117] Wentzel KR (2002). Are effective teachers like good parents? Teaching styles and student adjustment in early adolescence. Child Development.

[CR118] Wikstrom P-O, Svensson R (2010). When does self-control matter? The interaction between morality and self-control in crime causation. European Journal of Criminology.

[CR119] Willems F, Denessen E, Hermans C, Vermeer P (2012). Students’ perceptions and teachers’ self-ratings of modelling civic virtues: An exporatory empirical study in Dutch primary schools. Journal of Moral Education.

[CR120] Wolfe SE (2011). The effect of low self-control on perceived police legitimacy. Journal of Criminal Justice.

[CR121] Wolfe SE, McLean K, Pratt TC (2017). I learned it by watching you: Legal socialization and the intergenerational transmission of legitimacy attitudes. British Journal of Criminology.

[CR123] Wu Y, Lake R, Cao L (2015). Race, social bonds, and juvenile attitudes toward the police. Justice Quarterly.

[CR124] Young JTN, Rees C, Gibson CL, Krohn MD (2013). Social networks and delinquency in adolescence: Implications for life-course criminology. Handbook of life-course criminology: Emerging trends and directions for new research.

